# Severe Motor Deterioration of Young-Onset Parkinson's Disease in Pregnancy Following Medication Rationalisation: A Case Report

**DOI:** 10.7759/cureus.119359

**Published:** 2026-11-09

**Authors:** Ei Ei P Naing, Naw Phu N Ko Ko, Mya K Phyu

**Affiliations:** 1 Obstetrics and Gynaecology, Luton and Dunstable University Hospital, Luton, GBR; 2 Obstetrics and Gynaecology, Queen Elizabeth Hospital King's Lynn, Kings Lynn, GBR

**Keywords:** amantadine, caesarean section, dyskinesia, levodopa, multidisciplinary care, pregnancy, young-onset parkinson's disease

## Abstract

Parkinson's disease (PD) in pregnancy is rare, and there are no agreed guidelines for its management. We report a 34-year-old primigravida with a three-year history of young-onset PD who presented at eight weeks' gestation with an unplanned pregnancy. She had previously tolerated levodopa poorly, with nausea and early peak-dose dyskinesia, and before conception her symptoms were reasonably controlled on amantadine, selegiline and procyclidine. Because of the potential teratogenicity of amantadine and limited safety data for the other agents, her treatment was changed to co-careldopa (carbidopa/levodopa) with domperidone.

Her condition deteriorated soon after the switch. Despite stepwise increases in levodopa to 400-450 mg/day, by 31 weeks she was wheelchair-dependent, with severe peak-dose dyskinesia alternating with "off" periods and freezing, and she relied on her husband for feeding and personal care. Other drug options and deep brain stimulation were deferred until after delivery. Her care involved neurology, obstetric medicine, obstetric anaesthesia, safeguarding midwives, physiotherapy and occupational therapy, and she received thromboprophylaxis for immobility.

She presented in spontaneous labour at 37+5 weeks and underwent a category 2 caesarean section under general anaesthesia, as constant tremor precluded neuraxial anaesthesia. Estimated blood loss was 750 mL. Her daughter had Apgar scores of 4, 7 and 9 at 1, 5 and 10 minutes, respectively, and weighed 2250 g (3.4th centile). Postpartum, dyskinesia and tremor temporarily prevented her from holding her baby, and trihexyphenidyl was started; her symptoms were unchanged five weeks after delivery. In young-onset PD, withdrawal of an effective pre-pregnancy regimen may be followed by significant functional decline, and previous levodopa intolerance may be a warning sign. This case highlights the importance of pre-conception counselling, early multidisciplinary planning and postnatal support.

## Introduction

Parkinson's disease (PD) is the second most common neurodegenerative disorder, but onset during the reproductive years is uncommon, and pregnancy in women with PD is rare [1,2]. Young-onset PD, also termed early-onset PD, refers to onset between the ages of 21 and 50 years [3]. With increasing maternal age and better recognition of young-onset disease, obstetricians are likely to encounter it more often [4].

Younger patients are particularly prone to levodopa-related motor complications. These include 'wearing-off' fluctuations, in which symptoms return before the next dose is due, and dyskinesia, which are involuntary movements at peak drug levels. For this reason, treatment in young-onset PD often includes levodopa-sparing drugs, such as monoamine oxidase B (MAO-B) inhibitors and anticholinergics, as well as amantadine, the main oral treatment for dyskinesia. Pregnancy raises concerns beyond teratogenicity, including changes in drug absorption and distribution, the suppression of prolactin by dopaminergic drugs and its effect on breastfeeding, and the obstetric and anaesthetic implications of severe motor disability [4,5].

Most of the evidence on managing PD in pregnancy comes from case reports and small series. A systematic review identified 37 pregnant women with PD across 20 studies [1]. Young et al. found no standardised guidance, although clinicians generally favoured optimising motor control, using levodopa monotherapy and aiming for vaginal birth unless there was an obstetric indication otherwise [4]. Levodopa has the most reassuring safety record, whereas amantadine has been associated with fetal complications, including cardiac malformations [5,6].

The effect of pregnancy on motor symptoms is unpredictable. In a review of 79 cases, about 41% of women deteriorated, while 44% remained stable or improved [7]. Little has been reported on the effect of stopping an established multi-drug regimen in early pregnancy, particularly in women who have previously tolerated levodopa poorly [4,5]. We describe a woman with young-onset PD and previous levodopa intolerance whose condition declined markedly after amantadine, selegiline and procyclidine were replaced with levodopa in the first trimester, and we outline her antenatal, intrapartum and postnatal care.

## Case presentation

Background

A 34-year-old primigravida with young-onset PD was referred to a tertiary movement disorders clinic at eight weeks' gestation with an unplanned pregnancy. Her symptoms began three years earlier, at around 31 years of age, shortly after a bereavement, during which she was treated for low mood with antidepressants for approximately two years (agents and doses unknown). She developed a left-hand resting tremor with stiffness and slowness of movement, progressing to bilateral involvement. PD was diagnosed, and levodopa was started in her country of origin; she relocated to the United Kingdom about 18 months before conception.

A dopamine transporter (DaT) scan performed before referral reportedly showed reduced striatal uptake. The diagnosis of PD was made by her neurologists on clinical grounds, supported by the DaT scan and her initial response to levodopa; formal application of the MDS clinical diagnostic criteria [8] was not documented. Brain magnetic resonance imaging (MRI), investigations for Wilson's disease (serum caeruloplasmin, 24-hour urinary copper and slit-lamp examination), formal cognitive screening and genetic testing were not documented in the available records. Features favouring PD over atypical parkinsonism, including multiple system atrophy (MSA), were her initial response to levodopa, the development of levodopa-induced dyskinesia, normal eye movements on examination at 17 weeks and the absence of documented orthostatic hypotension. Her urinary incontinence was reported mainly when her medication was reduced or stopped.

She had initially responded well to levodopa, but the benefit was limited by nausea and peak-dose dyskinesia 15-20 minutes after each dose. Her husband described an earlier episode on co-careldopa when involuntary movements left her largely bedridden for about a month, and it had been tapered and stopped one month before the tertiary review. At conception, she was taking amantadine 100 mg in the morning and 200 mg at night, procyclidine 5 mg twice daily and selegiline 10 mg daily. On this regimen, she was independent in washing, dressing and feeding.

Non-motor symptoms included urinary incontinence, occasional dizziness and intermittent short-term memory difficulties. She denied hyposmia, hallucinations, constipation and symptoms of rapid eye movement (REM) sleep behaviour disorder. Her father was diagnosed with PD in his seventies, and her parents were not consanguineous. She had no other medical or surgical history and no known drug allergies. Genetic testing was not performed.

On examination at eight weeks, she had hypomimia, bilateral resting tremor (worse on the right), and bilateral rigidity and bradykinesia (worse on the left). Her gait was slow, with short steps, a stooped posture and en bloc turning. A right exotropia and a possible right cataract were also noted.

Antenatal course

After counselling, she and her husband agreed to switch to levodopa, given its more reassuring safety data in pregnancy. Domperidone 20 mg daily was started, followed three days later by co-careldopa 12.5/50 mg, increased from once to three times daily over one week. Selegiline was stopped, amantadine was tapered over two weeks, and procyclidine was withdrawn. Because of a delay in receiving written instructions, she had only just started co-careldopa by her next review at around 12 weeks.

At this point, she and her husband felt she was about 20% worse. She was markedly rigid, unable to turn in bed or while walking, and no longer able to prepare meals. Levodopa was increased in stages (Table 1). At 17 weeks, there was a modest improvement in rising from a chair, but this was not sustained. No formal rating scales (MDS Unified Parkinson's Disease Rating Scale (MDS-UPDRS) part III, Hoehn and Yahr or Schwab and England) were recorded during pregnancy; motor and functional status was documented descriptively by the neurology, obstetric and therapy teams.

**Table 1:** Antiparkinsonian treatment and motor status during pregnancy and the puerperium TDS - three times daily; QDS - four times daily

Gestation	Treatment	Levodopa (mg/day)	Motor and functional status
Pre-conception	Amantadine 300 mg/day, procyclidine 10 mg/day, selegiline 10 mg/day	0	Independent in activities of daily living
8 weeks	Switch to co-careldopa 12.5/50 mg planned (target TDS) with domperidone; adjuncts tapered	0 (target 150)	Bilateral tremor, rigidity, bradykinesia; en bloc turning
About 12 weeks	Co-careldopa started, then titrated to 2 tablets TDS over 3 weeks	300	Approx. 20% worse; unable to turn or cook
17 weeks	Increased to 3 tablets TDS	450	Modest improvement; still undertreated, hypophonia
31 weeks	Redistributed to four doses (2-2-2-3 tablets)	450	Severe peak-dose dyskinesia alternating with "off" periods
32+4 weeks	2 tablets QDS, plus 1 extra tablet if very stiff	400-450	Wheelchair-dependent; freezing; husband feeding her
34+1 weeks	Unchanged	400-450	No improvement; extra dose worsened tremor
Postpartum	Co-careldopa 25/100 mg continued (dose not recorded); trihexyphenidyl 1 mg TDS added, titrating	Not recorded	Transient inability to hold baby; mobile with hand-held assistance

Tablets refer to co-careldopa 12.5/50 mg (50 mg levodopa per tablet) unless stated. Levodopa equivalent daily dose before conception was approximately 400 mg (amantadine 300 mg; selegiline 10 mg, equivalent to 100 mg) [9]. No formal motor rating scales were recorded.

At booking (15+1 weeks), her body mass index (BMI) was 28.6 kg/m² and blood pressure 115/77 mmHg. Infection serology and haemoglobinopathy screening were normal, and other laboratory results during pregnancy and after delivery are summarised in Table 2. She was managed jointly by the obstetric and neurology teams, with serial growth scans from 28 weeks. The obstetric plan at booking was to aim for vaginal birth unless an obstetric indication for caesarean section arose. The anomaly scan at 18+5 weeks showed a low-lying placenta, which was no longer low-lying on follow-up scans, and was otherwise normal. A 75 g oral glucose tolerance test (OGTT) at 26 weeks and urinary protein and albumin measurements were normal. Haemoglobin fell to 117 g/L at 20 weeks, and oral iron was started. Growth scans at 28, 32 and 36 weeks showed an estimated fetal weight between the 86th and 90th centiles, with normal liquor volume and umbilical artery Doppler.

**Table 2:** Laboratory investigations during pregnancy and after delivery OGTT - oral glucose tolerance test

Investigation	Result (timing)	Reference range
Haemoglobin (g/L)	132 (booking); 117 (20 weeks); 121 (day of delivery); 111 (after delivery)	≥110 (first trimester); ≥105 (second and third trimesters); ≥100 (postpartum)
Platelets (×10⁹/L)	363 (booking); 279 (20 weeks); 252 (day of delivery); 241 (after delivery)	150–400
White cell count (×10⁹/L)	12.1 (day of delivery); 13.2 (after delivery)	4.0–11.0
Glycated haemoglobin (HbA1c) (mmol/mol)	31 (booking)	<42
OGTT fasting glucose (mmol/L)	4.1 (26 weeks)	<5.6
OGTT 2-hour glucose (mmol/L)	5.6 (26 weeks)	<7.8
Urine protein:creatinine ratio (mg/mmol)	22; 19	<30
Urine albumin:creatinine ratio (mg/mmol)	0.8; 1.7	<8

The obstetric anaesthetic team planned for both vaginal and caesarean birth. For labour, they recommended early epidural analgesia and discussed the risk of orthostatic hypotension. They advised avoiding pethidine, tramadol and haloperidol, with ondansetron or cyclizine as safe antiemetics. For caesarean section, they recommended fluid preloading, left lateral tilt and early vasopressors. They also noted an increased risk of aspiration under general anaesthesia because of dysphagia, the onset of which was not documented, and possible gastroparesis, and emphasised that her Parkinson's medication should not be interrupted around delivery.

By 31 weeks she had severe, disabling peak-dose dyskinesia, described by the therapy team as writhing movements, and became very stiff and "off" as each dose wore off. Dopamine agonists, alternative levodopa formulations and assessment for deep brain stimulation (DBS) were deferred until after pregnancy. At a multidisciplinary clinic at 32+4 weeks, attended by her consultant obstetrician, maternal medicine consultant and neurology consultant from the local hospital, she presented in a wheelchair. Freezing prevented her from turning while walking, and she had a shuffling gait with bilateral rigidity and tremor. Her husband was feeding her and assisting with personal care. Blood pressure was 98/67 mmHg, with no postural symptoms. The team's assessment was undertreated PD causing severe functional impairment.

Prophylactic tinzaparin 4500 units daily was started because of her immobility, and she was advised to take levodopa 30 minutes before meals. Because of her worsening tremor and inability to rest, the multidisciplinary team decided on an elective caesarean section at 38-39 weeks. Postpartum, opioids were to be avoided, with paracetamol and ibuprofen used for analgesia. She was counselled that returning to amantadine or procyclidine after delivery would probably be incompatible with breastfeeding, and she was referred to the infant feeding team. Safeguarding midwives were involved because of concerns about her ability to care for a newborn, and the couple were also facing the loss of their accommodation. At 34+1 weeks, there had been no improvement. The obstetrics theatre team, obstetrics anaesthetists, obstetrics consultants and midwifery teams were informed in advance of the planned elective caesarean section to allow appropriate planning for moving and handling.

Delivery

At 37+5 weeks, before the planned date, she presented in spontaneous labour and underwent a category 2 caesarean section. Constant tremor made neuraxial anaesthesia unfeasible, so the procedure was performed under general anaesthesia. Estimated blood loss was 750 mL, and no transfusion was required; haemoglobin before and after delivery is shown in Table 2. Her daughter weighed 2250 g (3.4th centile), with Apgar scores of 4, 7 and 9 at 1, 5 and 10 minutes, respectively. This was lower than predicted by the growth scans, and the reason for the discrepancy is unclear. The baby was admitted to the special care baby unit for one day and then discharged home.

Postnatal course

On the first postnatal day, worsening dyskinesia and tremor meant she was unable to hold her baby. The following day she was calmer, with only a mild right-hand tremor, and had supervised skin-to-skin contact. She was able to stand from the bed and walk across the room with two-handed support from her husband. The neurology team found severe parkinsonism with bradykinesia, dystonia and tremor, and started trihexyphenidyl 1 mg three times daily, with each dose increased by 2 mg every three days as tolerated. The reason for not restarting the full pre-conception regimen at this stage was not documented. Antenatally, amantadine and procyclidine had been noted to be incompatible with breastfeeding, which she hoped to do, and further treatment changes were deferred to her tertiary movement disorders review.

She was discharged five days after delivery on co-careldopa 25/100 mg tablets, trihexyphenidyl, tinzaparin and laxatives, with neurology follow-up in four weeks. The postnatal co-careldopa dose and the reason for the change from 12.5/50 mg to 25/100 mg tablets were not recorded in the discharge summary. A safe discharge required emergency housing arranged by the safeguarding midwives, a package of care, an occupational therapy assessment for equipment and a wheelchair, and an agreement that her husband would lift and carry the baby. Although she had wished to breastfeed, the baby was bottle-fed at discharge; the specific reason for this decision was not recorded. At telephone follow-up five weeks after delivery, her symptoms were unchanged. She was taking trihexyphenidyl 4 mg three times daily, and her daughter was well and bottle-fed. She is awaiting review in the tertiary movement disorders clinic, where the treatment options deferred during pregnancy will be reconsidered.

## Discussion

Within six months of switching to levodopa monotherapy, our patient progressed from independent living to wheelchair dependence. Prioritising fetal safety is an appropriate starting point when prescribing in pregnancy, but in this case it came at considerable cost to the mother.

Medication rationalisation

Among antiparkinsonian drugs, levodopa has the most human pregnancy data, and expert opinion favours its use as monotherapy [1,4]. Published reviews have not identified major malformations or birth complications directly attributable to levodopa at doses of up to 2500 mg/day [5,7]. Amantadine, in contrast, is teratogenic in animals and has been associated with cardiac malformations in humans, and in early reviews most complicated pregnancies involved amantadine [2,6]. Data on selegiline and anticholinergics are limited [2,4]. Discontinuing amantadine in the first trimester was therefore a reasonable decision.

In retrospect, however, there were indications that the transition might be difficult. She had previously tolerated levodopa poorly, with nausea and early, disabling dyskinesia. Amantadine is often used specifically to reduce levodopa-induced dyskinesia, so its withdrawal removed the drug best suited to her main side effect. Her levodopa equivalent daily dose (LEDD) was similar before conception (approximately 400 mg) and during pregnancy (400-450 mg) [9], suggesting that the change in drug class, rather than the total dose, may have been important. Her therapeutic window became very narrow: doses high enough to relieve rigidity and freezing produced severe dyskinesia, and an additional tablet worsened her tremor.

Pregnancy itself may also have contributed. Around 40% of women with PD experience worsening of symptoms during pregnancy, and in some, the deterioration persists postpartum [7,10]. Proposed mechanisms include changes in drug absorption and distribution, delayed gastric emptying and fluctuating hormone levels [1,5]. The delay in starting co-careldopa, which left her with little dopaminergic treatment for several weeks while amantadine and selegiline were being withdrawn, may also have contributed. In a single case, it is not possible to separate the effects of pregnancy, the medication change, the delay in starting levodopa and disease progression.

Options to improve her motor control were limited. Dopamine agonists have less pregnancy safety data than levodopa [4], and DBS, although reported in pregnant women with PD [4], was deferred. Had the pregnancy been planned, the medication change could have been made under supervision, or DBS considered, before conception. This highlights the importance of early discussion of contraception and pre-conception planning with women of reproductive age who have PD.

Intrapartum and anaesthetic care

Vaginal birth is generally recommended for women with PD [4], and this was the initial plan at booking. However, at 32+4 weeks the multidisciplinary team recommended elective caesarean section because of her worsening tremor and inability to rest. Continuous involuntary movement was expected to make labour physically exhausting and to interfere with positioning, continuous fetal monitoring and the siting of an epidural. A planned delivery also allowed her antiparkinsonian medication to be timed and continued around surgery. In the event, she presented in spontaneous labour at 37+5 weeks, before the planned date, and the operation was performed as a category 2 procedure. Several anaesthetic concerns were anticipated. Tremor and dyskinesia can make neuraxial techniques difficult, autonomic dysfunction increases the risk of hypotension, dysphagia and delayed gastric emptying increase aspiration risk under general anaesthesia, and dopamine antagonists such as haloperidol can cause acute deterioration [11]. At delivery, her continuous tremor made neuraxial anaesthesia unsafe. The baby's low 1-minute Apgar score with rapid recovery is consistent with transient neonatal depression following general anaesthesia. Pethidine and tramadol were avoided because of their serotonergic effects, which are particularly relevant if an MAO-B inhibitor is restarted. Equally important, missed or delayed levodopa doses around surgery can cause severe rigidity, so a written medication plan and early resumption after delivery are essential.

Thromboprophylaxis, postnatal care and social context

Her immobility added to the thromboembolic risk of late pregnancy and caesarean section, and she received low-molecular-weight heparin both antenatally and postnatally. After delivery, the main priority was safe handling of the baby. The physiotherapy, occupational therapy and midwifery teams agreed with the couple that her husband would lift and carry the baby and that her contact with the baby would be supervised.

Medication also influenced infant feeding. Amantadine is not recommended during breastfeeding, and anticholinergics such as trihexyphenidyl may reduce milk supply, so decisions about postnatal treatment and feeding were closely linked. Trihexyphenidyl was started after delivery, and although she had wished to breastfeed, the baby was bottle-fed at discharge. Her care also depended heavily on safeguarding midwives, emergency housing and a package of care. For women with disabling neurological conditions, social and practical support in pregnancy requires the same careful planning as medical management.

This report has limitations. The diagnostic workup was incomplete: brain MRI, screening for Wilson's disease and genetic testing were not documented, so a secondary or genetic cause of young-onset Parkinsonism cannot be excluded, particularly given her family history. No formal motor rating scales were recorded, so changes in her motor status are based on clinical descriptions. Follow-up after delivery was limited to five weeks.

## Conclusions

Levodopa monotherapy remains the preferred treatment for PD in pregnancy. However, in this patient, withdrawal of an effective multi-drug regimen was followed by severe deterioration that persisted five weeks after delivery, and previous levodopa intolerance or dyskinesia may be a warning sign. Women with PD who may become pregnant should be offered pre-conception counselling and a planned medication strategy. During pregnancy, early multidisciplinary planning involving neurology, obstetrics, anaesthesia, midwifery, therapy and social care is important, covering thromboprophylaxis, uninterrupted peripartum medication and support for infant care and feeding.
